# Can body-mapped garments improve thermal comfort for sport in the cold?

**DOI:** 10.1186/2046-7648-4-S1-A74

**Published:** 2015-09-14

**Authors:** Damien Fournet, Bernard Redortier, George Havenith

**Affiliations:** 1 Thermal Sciences Laboratory, Decathlon SportsLab, Villeneuve d'Ascq, France; 2 Environmental Ergonomics Research Center, Loughborough University, Loughborough, UK

## Introduction

Body maps of the human body have been developed regarding distribution of sweat production [1] and skin temperature [2]. However it has rarely been explored how body-mapped clothing (textile properties adjusted locally according to body mappings) impact thermal responses and perceptions. The present study investigates the question for exercising in the cold, manipulating skin temperatures by various distributions of clothing. It was hypothesized that covering naturally cold regions would make skin temperature more uniform and be beneficial for thermal comfort.

## Methods

Twelve physically active males performed a 40 min running bout (70% $\overset{\cdot }{\text{V}}{\text{O}}_{\text{2max}}$) followed by 10min recovery at 5 °C, 70%rh wearing three different clothing ensembles having similar total insulation, but with local insulation varying according to temperature mapping (Fournet et al., 2013). Clothing **U **had uniform insulation all over the body; clothing **C **extra insulation on cold regions and less on warm regions; clothing **W **was the opposite of **C**, with extra insulation on warm regions. Skin temperatures (T_sk_) were recorded by infrared thermography, with calculation of mean T_sk _and T_sk _variability (T_sk _uniformity across the body surface). Rectal temperature (T_re_), heart rate (HR), oxygen uptake ($\overset{\cdot }{\text{V}}{\text{O}}_{2}$) were measured as well as thermal sensation and comfort (Likert scales).

## Results

All clothing designs provided similar mean T_sk _through the protocol (**C**: 25.7 ± 1.7 °C, **W**: 25.9 ± 1.6 °C, **U**: 25.3 ± 1.7 °C, NS), with larger T_sk _variability for **W **compared to **C **and **U **(2.4 °C vs 1.8 °C and 2 °C, p < 0.05). The largest regional T_sk _differences were measured on the anterior torso and legs (+2.5°C and +2.6 °C for **C **vs **W**) and the upper back (+3 °C for **W **vs **C**) highlighting the influence of extra insulation. No significant differences were observed neither on T_re _(37.9 °C at 40 min in **C**, **W**, **U**), nor on HR or $\overset{\cdot }{\text{V}}{\text{O}}_{2}$. Overall thermal sensation and comfort votes were similar between conditions after 40 min of running. Following recovery, participants wearing **C **had small but significantly improved perceptions compared to **U **(*neutral *vs *slightly cool *p < 0.05; *comfortable *vs *slightly uncomfortable *p < 0.1). The effect of clothing design on local thermal sensation was significant for 5 out of 11 body regions (with varied insulation) but negligible in terms of local thermal comfort (only larger discomfort at the back at 40 min for **W **vs **C**).

## Discussion and conclusion

Nielsen and Nielsen [3] found that insulating upper vs lower body (no fine bodymapping) induced similar thermal sensations despite different T_sk _distribution. Mean T_sk _was however also different. Our clothing intervention was successful in controlling for mean T_sk _by manipulating regional T_sk_. Covering specific cold regions (i.e reducing T_sk _variability) did not provide a thermoregulatory or a decisive perceptual advantage. Though sweat production was limited during exercise, evaporative heat loss was not favoured due to the extra insulation and this, in turn, amplified moisture perception. At this exercise intensity, optimal insulation with evolutive (on/off) openings could be preferable for comfort maintenance.
